# Dancing with Gravity—Why the Sense of Balance Is (the) Fundamental

**DOI:** 10.3390/bs8010007

**Published:** 2018-01-05

**Authors:** Dominik Fuchs

**Affiliations:** Research Centre Allgäu (FZA), University of Applied Sciences Kempten, 87435 Kempten, Germany; dominik.fuchs@hs-kempten.de; Tel.: +49-831-2523-148

**Keywords:** balance, vestibular system, embodiment, cognition, arts

## Abstract

The sense of balance, which is usually barely noticeable in the background of each of our movements, only becomes manifest in its function during intense stimulation or in the event of illness, which may quite literally turn your world upside down. While it is true that balance is becoming a bigger issue, that is mainly because people are losing it more frequently. So why is balance not as commonly talked about in psychology, medicine or the arts as the other five traditional senses? This is partly due to its unusual multi-modal nature, whereby three sensory inputs are coordinated and integrated by the central nervous system. Without it, however, we might not have much use for the other senses. The sense of balance encompasses the bodily experience in its entirety. Not only do we act with the body, we may also think and feel through it and with it. Bodily states are not simply effects of cognition; they cause it as well. Equilibrioception is an essential sense and it is interconnected with a wide range of other areas, including cognition, perception, embodiment, the autonomic nervous system, aesthetics, the arts, and education.

## 1. Metaphorical Balance

One of the reasons that Hillary Clinton lost her staggering two-digit lead over Donald Trump in the 2016 United States presidential election was that she was repeatedly seen staggering in public [1]. Her stumbling was apparently conceived by millions as a sign of weakness, of unreliability. She appeared to be *off balance*.

Just how important physical balance is for people can be seen in the ubiquity of the talk of “balance”. We use the word “balance” in a multitude of domains: Work-life balance, account balance, mental balance. Balance of interest, power, risk, and terror. Physical experience is projected to more remote and abstract areas, showing how parts of our thinking are based on body experiences and not on rules or concepts [2,3]. As Lakoff and Johnson pointed out, metaphors or “image schemas” like balance have a strong influence on our perception, thoughts, feelings, and actions [2,4,5,6].

Despite the growing literature on balance, what the present opinion piece aims to illustrate is that the actual *sense* of balance is still underrated compared to the other traditional senses and given that it is an essential foundation for numerous physical and cultural phenomena. One of the reasons for this underestimation is that its main sensory input, the vestibular apparatus, is arguably the most mysterious sensory organ in the body: (1) It acts unobtrusively; among the sensory systems, the equilibrium system is the one that is least consciously perceived under physiological conditions [7,8]. (2) How the relating information is processed, and in which functional circuits it is integrated, is not yet fully explored [9,10]. You won’t understand perception by merely exploring the sense organs. Trying to understand static and dynamic balance by studying the individual sensory organs is like trying to understand a traffic jam by looking inside the internal combustion engines.

## 2. Physiology and Characteristics of the Sense of Balance

**Figure 1:**
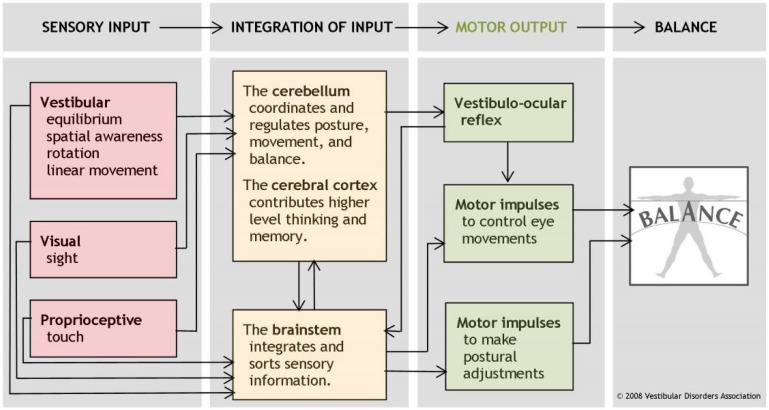
The human balance system. Courtesy of the Vestibular Disorders Association (vestibular.org).

Generally speaking, balance is the ability to control one’s own center of gravity in relation to the support area in order to maintain an upright posture (Figure 1) [11]. It has the function of controlling a body position in space, and is therefore an essential prerequisite for sitting, standing, walking, and virtually every other activity of everyday life [12]. Accordingly, in neurologic patients, postural control is the best predictor of regaining an independent life after rehabilitation [11].

Balance is becoming a bigger medical and public issue, mainly because people are losing it more frequently due to demographic changes [13,14]; we usually do not think about it until it is impaired. If you raise your hand, you *know* that your arm is over your head. With bilateral vestibulopathy, for instance, you now have to *think* where you are in space or how you get from here to there [15,16].

The sensory modality of balance is not like hearing (sound) or seeing (light) clearly attributable to a sense organ. But if you take a closer look at other senses, it becomes clear that they are not as neatly separated from each other as we assume them to be based on our everyday experience [3]. Even with the usually dominant vision, through the vestibulo-ocular reflex, your eyes adjust themselves based on balance information—that’s how eyes adapt to your movement (Figure 1). This reflex has a latency of only 8 ms; by comparison, the visual system would take about 10 times as long to detect a shift of the image on the retina and to initiate compensatory movements of the head [7]. In this broader sense, balance information is also part of the visual process—and an example of the multisensory nature of our perception at large.

It is said that (perfect) balance is the action of not moving [17]. Well, no one can *really* stand still, because postural control involves constant nearly imperceptible movements and the integration of many different sensory inputs [13,15,18]. But although we usually take it for granted and give it little thought, you would probably agree that it is quite helpful to be able to maintain an upright posture—or “to stand despite all possibilities to fall” [19].

The balancing act is much more than a skillful mini-muscle game, though. Proprioceptive perception or deep-sensibility is the ability to sense what is going on in joints, muscles, tendons. And in addition to the visual and tactile sensory input, especially the vestibular organs in the inner ear are responsible for our equilibrium (Figure 1) [13]. Inside is what is appropriately called the labyrinth—hoses, liquids, sacks, pebbles, and sensory hairs that we use to detect movement and acceleration. If there is a malfunction somewhere, dizziness, disorientation, injuries or even cognitive and mental problems occur (which will be further illustrated below) [8,18,20,21]. For true independence, we depend on our balance. Balance is the bodily *condicio sine qua non*.

To still claim we only have five senses is a bit outdated (sorry, Aristotle [22]). The fact that balance was not listed in these “big five” is partly due to its unusual composition. It’s strictly speaking not *one* sense, but combined sensory information [7,15,18]. Depending on the context, the requirements for the mentioned inputs are different [13]. Standing on firm ground is different than standing on a surf board [12]. The otolith organs, for instance, cannot distinguish whether you are sitting in an accelerating bus or you are lying on your back. For this, you need additional information from the semicircular canals or your eyes [9].

## 3. The Vestibular System and Embodied Cognition

It is well known that through what is called cross-modal interaction, a visual event can change the perception of a sound and vice versa (e.g., ventriloquist) [23,24]. But that pertains to balance and posture as well; for example, leaning to the left makes the Eiffel Tower look smaller [25]. Findings like these provide further evidence for balance-related phenomena of embodiment or embodied cognition, which states that the content of the mind is partly determined by the body [26]. Posture may also play an important role for emotional states; in a recent study, stooped posture led to and preserved a negative mood (compared to straight posture) [27]. Another study has shown that people who are experiencing internal ambivalence move from side to side more than others, and when people are made to move from side to side, their experiences of ambivalence increase [28].

Patients with vestibular disorders often report that they also have problems moving in their thinking (e.g., sequential tasks and multitasking), and their imagination [8,15]. Conversely, imagining—not perceiving—a target stimulus can influence the vestibulo-ocular reflex [9], which further illustrates that the vestibular system is connected to cognitive processes and not merely reflexive. These cognitive interactions include memory [29], attention [30], mental imagery [31,32], body awareness [33], and social cognition [34,35].

Caloric (CVS) and galvanic (GVS) vestibular stimulation are inexpensive and non-invasive techniques that can be used to stimulate the vestibular receptors (e.g., for clinical diagnosis or as a therapeutic method in neurological rehabilitation) and to investigate vestibular interactions with cognitive processes [10,16,36,37]. With CVS—the flushing of the external ear canal with water—and GVS—applying small-amplitude currents to the vestibular nerve—symptoms of somatoparaphrenia and neglect could be temporarily reduced, and mood and affective control could be modulated [8,9,38,39,40], providing evidence that vestibular stimulation can influence the self-perception of the body. GVS could also be used for commercial and military purposes; researchers of the Nippon Telegraph & Telephone Corp., Japan’s top telephone company (Tokyo, Japan), said they could steer a person along a winding route with GVS as if they had a remote control [41].

We are only just beginning to understand how the body influences mental function, but it now seems quite clear that the influence of the body extends to abstract concepts (e.g., through embodied and ingrained metaphors that help us to conceptually structure our experience [2,5]) and—as research shows—even to complex cognitive processes such as decision-making [42,43].

## 4. Spatial Awareness and Educational Implications

Several recent studies have explored connections between spatial abilities and mathematics [44,45]. For instance, higher levels of spatial sense predict stronger performance on standardized mathematics tests [46]. The process of postural control also includes the ability to orient oneself, that is, to maintain a relation between body segments on the one hand, and between the body and gravity/the environment on the other hand [12]. The vestibular system includes parts of the inner ear and brain that help control spatial awareness and orientation—which in turn are weakened when vestibular input is lacking [21,47,48]. So, from a developmental point of view, vestibular information and spatial skills are a central aspect of evolutionary adaptation with obvious practical significance—and a key component of human intellect [48,49]. The vestibular system appeared more than 500 million years ago to detect gravitational vertical [50], which makes it more than likely that our central nervous system developed a certain dependence on it [51].

Although recent findings suggest that there is more to thinking and learning than previously supposed, teaching in schools is still largely designed to train only the mind [52]. However, language, for instance, is not only processed in the classic cortical “language areas”, but also in areas which control movement [53,54]. Even higher-level cognition is rooted in bodily awareness, which therefore needs to be nurtured, for it is a mainstay of intellectual reasoning [52].

Body and brain work together in a “brain-body” system [55]. This concept can offer a new orientation of school education. Scientifically grounded physical, visual, and auditory intervention elements have led to new promising educational approaches [52]. At a time when artistic disciplines and motor learning are increasingly marginalized in favor of mathematical, scientific, and language-related skills, this research is of considerable importance for the rehabilitation of artistic and physical education [56].

## 5. Balance, Arts and Aesthetics

The sense of equilibrium is a musical sense. Not only are its main contributors seated in the (inner) ear and its signals transferred through the same cranial nerve as auditory signals (the auditory vestibular nerve) [57], an acoustic sensitivity of the vestibular apparatus has also been well established [58]. Both in the auditory and the vestibular domain, dominant themes are: distance, directions, height and depth, space, orientation. Recent studies have shown a correlation between a form of amusia (the inability to distinguish pitch) and poor spatial perception [59,60]. Brain research points out that pitch distinctions and spatial imagination largely occupy the same brain regions [56].

And just like the fundamental of a harmonic musical structure, balance provides a decent and dependable foundation and reference point for the other senses to fully unfold. In balance and music, rhythm is a basic element, it structures the experience; when you walk, you rhythmically move in time like a swinging metronome—the more harmonious the movement, the better the walk looks (and sounds).

Balance rocks—a chair, a baby, a boat—and it likes dancing. Dance—the playful administration of body movement and posture—goes beyond visual or spatial balance. It becomes experiential; a risky and rhythmical surrender and re-gaining of balance. People are moving and constantly see others moving. But when we dance, balance enables us to exceed the habitual and enter a state of dynamic equilibrium between lability and stability [61], often accompanied by interpersonal resonance phenomena (mainly through rhythm) [62,63].

**Figure 2:**
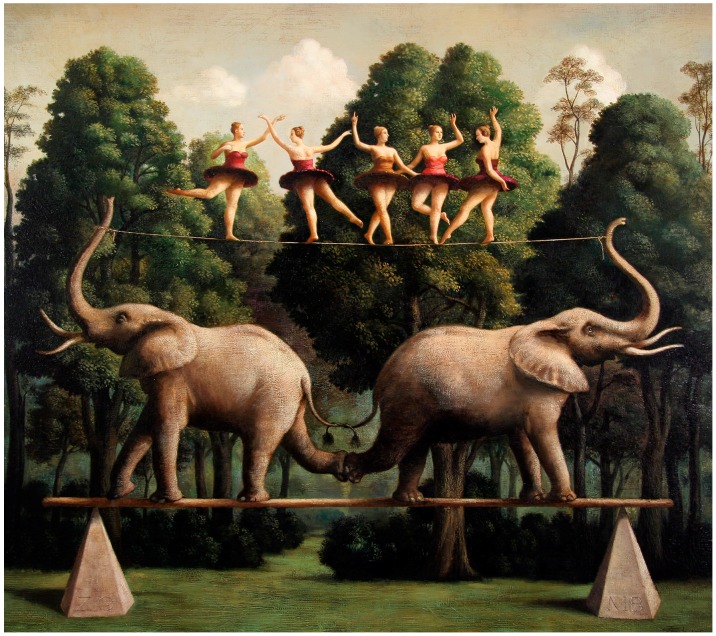
“Art of Balance” (2006). Painting by Ilya Zomb (http://www.zombart.com). The image displays both metaphorical and performed balance.

In the history of aesthetics, the individual senses have received very different attention. Although metaphorical balance is present in many pieces of art, e.g., as perceived symmetry (Figure 2), the physiological sense of balance barely attracted attention. If you define aesthetics as the science of the beautiful and the arts, then the focus lies on sensory modalities that can be artistically transformed as image, sound and shape, and you neglect all those sensual experiences, which—like the sense of balance—are considered to be less capable of art [61]. If balance is to be given attention in aesthetics, then one has to understand aesthetics as the science of sensory cognition (as Alexander Baumgarten defined it), too [6,61].

One of the oldest concepts of aesthetics is called “organic unity”, which is the idea that a thing or a piece of art is made up of interdependent parts, very much like the sense of balance is made up of its constituent sensory impressions [64].

## 6. Kinetosis and Vestibular Interactions with the Autonomic Nervous System

Another conundrum regarding the sense of equilibrium is motion sickness (or kinetosis); being probably the most common form of balance disorder [65], it still remains an enigmatic phenomenon. Although it is called *sickness*, its origin is not the stomach, but the senses [15]. While we know (for not too long) that it is mainly caused by a sensory conflict (e.g., the vestibular information about your movement does not match with the information from your eyes) [7], there is still a lack of research regarding its (evolutionary) cause and most adequate treatment [15].

Motion sickness and vertigo point to the connection of the vestibular sense and our autonomic nervous system, which in and of itself is a bodily system that relies on balance (between its sympathetic and parasympathetic branches [66]). If a patient complains of dizziness, the doctor will probably measure his pulse and blood pressure (functions that are regulated by the autonomic nervous system). Vestibular information is important for the control of blood pressure during movement and changes in posture [67]. There is also a special type of dizziness that is associated with poor autonomic regulation, called autonomic dizziness [68,69]. Yet, there must be something enticing about dizziness, since people frequently put considerable effort to experience its symptoms (e.g., rides, fair). In case of more severe impairments of the vestibular system, however, we should consider ourselves lucky to have brains with the extraordinary ability to compensate for weakness or dysfunction in any one of the sensory inputs by increasing the compensatory input of others [7].

## 7. Conclusions

So, on balance, equilibrium arguably does not receive its deserved cultural and physiological appreciation yet. That being said, there appears to be a growing popularity of the field coming both from trend sports and health care. Young people train their balance in slacklining, parkour or with a yoga board; elders practice it more seriously on adult playgrounds and in specific courses [20]. Furthermore, an increasing number of studies from fields like spatial [21] and social [34] cognition indicate a growing interest in the vestibular sense in the scientific community [10]. These progressions will desirably lead to an augmented awareness that the sense of balance is just as fundamental for our movements as it is for our thinking and overall well-being.

## References

[1] Collinson S., CNN Hillary Clinton Stumbles—Will Her Campaign Follow?. http://edition.cnn.com/2016/09/11/politics/hillary-clinton-health-2016-election/index.html.

[2] Johnson M. (1987). The Body in the Mind. The Bodily Basis of Meaning, Imagination, and Reason.

[3] Schönhammer R., Schönhammer R. (2009). Der Gleichgewichtssinn in materieller Kultur und Ästhetik—Ein Überblick. Körper, Dinge und Bewegung: Der Gleichgewichtssinn in Materieller Kultur und Ästhetik.

[4] Lakoff G., Johnson M. (1994). Metaphors We Live By.

[5] Slingerland E.G. (2008). What Science Offers the Humanities. Integrating Body and Culture.

[6] Mittelberg I., Borkent M., Dancygier B., Hinnell J. (2013). Balancing Acts: Image Schemas and Force Dynamics as Experiental Essence in Pictures by Paul Klee and their Gestural Enactments. Language and the Creative Mind, Proceedings of the 11th Conceptual Structure, Discourse and Language Conference, Vancouver, BC, Canada, 17–20 May 2012.

[7] Jahn K., Schönhammer R. (2009). Der Gleichgewichtssinn und seine Störungen—Schwindel aus Sicht des Neurologen. Körper, Dinge und Bewegung: Der Gleichgewichtssinn in Materieller Kultur und Ästhetik.

[8] Lopez C. (2016). The vestibular system: Balancing more than just the body. Curr. Opin. Neurol..

[9] Mast F.W., Grabherr L., Schönhammer R. (2009). Mit dem Körper denken—Der Gleichgewichtssinn als fundamentale Selbsterfahrung. Körper, Dinge und Bewegung: Der Gleichgewichtssinn in Materieller Kultur und Ästhetik.

[10] Besnard S., Lopez C., Brandt T., Denise P., Smith P.F. (2015). Editorial: The Vestibular System in Cognitive and Memory Processes in Mammalians. Front. Integr. Neurosci..

[11] Geurts A.C.H., de Haart M., van Nes I.J.W., Duysens J. (2005). A review of standing balance recovery from stroke. Gait Posture.

[12] Hofheinz M., Mibs M., Elsner B., Mehrholz J. (2011). Balancetraining nach Schlaganfall. Neuroreha Nach Schlaganfall: 18 Tabellen.

[13] Mehrholz J. (2011). Neuroreha Nach Schlaganfall. 18 Tabellen.

[14] Iwasaki S., Yamasoba T. (2015). Dizziness and Imbalance in the Elderly: Age-related Decline in the Vestibular System. Aging Dis..

[15] McCredie S. (2007). Balance. In Search of the Lost Sense.

[16] Wuehr M., Decker J., Schniepp R. (2017). Noisy galvanic vestibular stimulation: An emerging treatment option for bilateral vestibulopathy. J. Neurol..

[17] Goddard S. (2005). Reflexes, Learning and Behavior. A Window into the Child’s Mind.

[18] Schädler S. (2016). Gleichgewichtsstörungen und Schwindel. Grundlagen—Untersuchung—Therapie.

[19] Klee P., Regel G. (1991). Kunst-Lehre. Aufsätze, Vorträge, Rezensionen und Beiträge zur bildnerischen Formlehre.

[20] Strohmaier B. Balanceakt: Warum es so Wichtig ist, das Gleichgewicht zu trainieren—WELT. https://www.welt.de/icon/fitness/article170530583/Warum-es-so-wichtig-ist-das-Gleichgewicht-zu-trainieren.html.

[21] Mast F.W., Preuss N., Hartmann M., Grabherr L. (2014). Spatial cognition, body representation and affective processes: The role of vestibular information beyond ocular reflexes and control of posture. Front. Integr. Neurosci..

[22] Aristoteles, Corcilius K. (2017). Über die Seele. De anima. Griechisch-Deutsch.

[23] Theeuwes J., van der Burg E., Olivers C.N.L., Bronkhorst A., Kramer A.F., Wiegmann D.A., Kirlik A. (2007). Cross-Modal Interactions between Sensory Modalities: Implications for the Design of Multisensory Displays. Attention: From Theory to Practice.

[24] Bertelson P., Aschersleben G. (2003). Temporal ventriloquism: Crossmodal interaction on the time dimension. 1. Evidence from auditory-visual temporal order judgment. Int. J. Psychophysiol..

[25] Eerland A., Guadalupe T.M., Zwaan R.A. (2011). Leaning to the left makes the Eiffel Tower seem smaller: Posture-modulated estimation. Psychol. Sci..

[26] Barsalou L.W. (2008). Grounded cognition. Ann. Rev. Psychol..

[27] Veenstra L., Schneider I.K., Koole S.L. (2017). Embodied mood regulation: The impact of body posture on mood recovery, negative thoughts, and mood-congruent recall. Cogn. Emot..

[28] Schneider I.K., Eerland A., van Harreveld F., Rotteveel M., van der Pligt J., van der Stoep N., Zwaan R.A. (2013). One way and the other: The bidirectional relationship between ambivalence and body movement. Psychol. Sci..

[29] Smith P.F., Geddes L.H., Baek J.-H., Darlington C.L., Zheng Y. (2010). Modulation of memory by vestibular lesions and galvanic vestibular stimulation. Front. Neurol..

[30] Figliozzi F., Guariglia P., Silvetti M., Siegler I., Doricchi F. (2005). Effects of vestibular rotatory accelerations on covert attentional orienting in vision and touch. J. Cogn. Neurosci..

[31] Péruch P., Lopez C., Redon-Zouiteni C., Escoffier G., Zeitoun A., Sanjuan M., Devèze A., Magnan J., Borel L. (2011). Vestibular information is necessary for maintaining metric properties of representational space: Evidence from mental imagery. Neuropsychologia.

[32] Falconer C.J., Mast F.W. (2012). Balancing the mind: Vestibular induced facilitation of egocentric mental transformations. Exp. Psychol..

[33] Ferrè E.R., Vagnoni E., Haggard P. (2013). Vestibular contributions to bodily awareness. Neuropsychologia.

[34] Deroualle D., Lopez C. (2014). Toward a vestibular contribution to social cognition. Front. Integr. Neurosci..

[35] Lopez C., Falconer C.J., Mast F.W. (2013). Being moved by the self and others: Influence of empathy on self-motion perception. PLoS ONE.

[36] Palla A., Lenggenhager B. (2014). Ways to investigate vestibular contributions to cognitive processes. Front. Integr. Neurosci..

[37] Zubko O., Wilkinson D., Langston D., Sakel M. (2013). The effect of repeated sessions of galvanic vestibular stimulation on target cancellation in visuo-spatial neglect: Preliminary evidence from two cases. Brain Inj..

[38] Preuss N., Hasler G., Mast F.W. (2014). Caloric vestibular stimulation modulates affective control and mood. Brain Stimul..

[39] Schmidt L., Keller I., Utz K.S., Artinger F., Stumpf O., Kerkhoff G. (2013). Galvanic vestibular stimulation improves arm position sense in spatial neglect: A sham-stimulation-controlled study. Neurorehabilit. Neural Repair.

[40] Schönherr A., May C.A. (2016). Influence of Caloric Vestibular Stimulation on Body Experience in Healthy Humans. Front. Integr. Neurosci..

[41] Kageyama Y. A Remote Control that Controls Humans. http://www.nbcnews.com/id/9816703/ns/technology_and_science-innovation/#.WkR8WN_ibct.

[42] Preuss N., Mast F.W., Hasler G. (2014). Purchase decision-making is modulated by vestibular stimulation. Front. Behav. Neurosci..

[43] McKay R., Tamagni C., Palla A., Krummenacher P., Hegemann S.C.A., Straumann D., Brugger P. (2013). Vestibular stimulation attenuates unrealistic optimism. Cortex J. Devot. Study Nerv. Syst. Behav..

[44] Mix K.S., Levine S.C., Cheng Y.-L., Young C., Hambrick D.Z., Ping R., Konstantopoulos S. (2016). Separate but correlated: The latent structure of space and mathematics across development. J. Exp. Psychol. Gen..

[45] Sella F., Sader E., Lolliot S., Cohen Kadosh R. (2016). Basic and advanced numerical performances relate to mathematical expertise but are fully mediated by visuospatial skills. J. Exp. Psychol. Learn. Mem. Cogn..

[46] Mix K.S., Cheng Y.-L., Benson J.B. (2012). The Relation between Space and Math. Advances in Child Development and Behavior.

[47] Brandt T., Schautzer F., Hamilton D.A., Brüning R., Markowitsch H.J., Kalla R., Darlington C., Smith P., Strupp M. (2005). Vestibular loss causes hippocampal atrophy and impaired spatial memory in humans. Brain J. Neurol..

[48] Wiener-Vacher S.R., Hamilton D.A., Wiener S.I. (2013). Vestibular activity and cognitive development in children: Perspectives. Front. Integr. Neurosci..

[49] Newcombe N.S., Uttal D.H., Sauter M., Zelazo P.D. (2013). Spatial Development. Body and Mind.

[50] Graf W., Klam F. (2006). Le système vestibulaire: Anatomie fonctionnelle et comparée, évolution et développement. C. R. Palevol.

[51] Smith P.F., Darlington C.L. (2013). Personality changes in patients with vestibular dysfunction. Front. Hum. Neurosci..

[52] McClelland E., Pitt A., Stein J. (2015). Enhanced academic performance using a novel classroom physical activity intervention to increase awareness, attention and self-control: Putting embodied cognition into practice. Improv. Sch..

[53] Hauk O., Johnsrude I., Pulvermüller F. (2004). Somatotopic Representation of Action Words in Human Motor and Premotor Cortex. Neuron.

[54] De Lafuente V., Romo R. (2004). Language Abilities of Motor Cortex. Neuron.

[55] Clark A. (2001). Being There. Putting Brain, Body, and World Together Again.

[56] Rittelmeyer C. (2012). Warum und wozu ästhetische Bildung? Über Transferwirkungen Künstlerischer Tätigkeiten. Ein Forschungsüberblick.

[57] Sanders R.D., Gillig P.M. (2010). Cranial Nerve VIII: Hearing and Vestibular Functions. Psychiatry.

[58] Todd N.P.M., Paillard A.C., Kluk K., Whittle E., Colebatch J.G. (2014). Vestibular receptors contribute to cortical auditory evoked potentials. Hear. Res..

[59] Douglas K.M., Bilkey D.K. (2007). Amusia is associated with deficits in spatial processing. Nat. Neurosci..

[60] Williamson V.J., Cocchini G., Stewart L. (2011). The relationship between pitch and space in congenital amusia. Brain Cogn..

[61] Allesch C.G., Schönhammer R. (2009). Gleichgewichtssinn, Balance und Ästhetik. Körper, Dinge und Bewegung: Der Gleichgewichtssinn in Materieller Kultur und Ästhetik.

[62] Koch S., Bergmann T. (2017). Hooked to Rhythm: Übergänge und gemeinsame Grundlagen von Musik und Bewegung. Musikther. Umsch..

[63] Koch S.C. (2014). Rhythm is it: Effects of dynamic body feedback on affect and attitudes. Front. Psychol..

[64] Goodman A. (1991). Organic unity theory: The mind-body problem revisited. Am. J. Psychiatry.

[65] Frejo L., Giegling I., Teggi R., Lopez-Escamez J.A., Rujescu D. (2016). Genetics of vestibular disorders: Pathophysiological insights. J. Neurol..

[66] Jänig W., Schmidt R.F., Thews G. (1989). Autonomic Nervous System. Human Physiology.

[67] Yates B.J., Bolton P.S., Macefield V.G. (2014). Vestibulo-sympathetic responses. Compr. Physiol..

[68] Pappas D.G. (2003). Autonomic related vertigo. Laryngoscope.

[69] Staab J.P., Ruckenstein M.J. (2007). Autonomic nervous system function in chronic dizziness. Otol. Neurotol..

